# Predictors of obstructive sleep apnea misclassification when using total bed time versus total sleep time

**DOI:** 10.1038/s41598-021-90818-y

**Published:** 2021-06-01

**Authors:** Wei Yang Lim, Kay Choong See

**Affiliations:** Division of Respiratory & Critical Care Medicine, Department of Medicine, National University Hospital, Singapore, Singapore

**Keywords:** Medical research, Respiratory tract diseases

## Abstract

Obstructive sleep apnea (OSA) is a highly prevalent condition worldwide. Untreated, it is associated with multiple medical complications as well as a reduced quality of life. Home sleep apnea tests are increasingly used for its diagnosis and evaluation of severity, but using total bed time rather than total sleep time may underestimate OSA severity. We aim to uncover the extent and predictors of OSA misclassification when using total bed time. A retrospective observational study was conducted using data from the sleep laboratory of the National University Hospital, Singapore, a tertiary hospital with 1200 beds. Misclassification of OSA was defined as any OSA severity that was less severe using total bed time versus total sleep time. Logistic regression was used to identify predictors of OSA misclassification. A total of 1621 patients were studied (mean age 45.6 ± 15.9 years; 73.4% male). 300 (18.5%) patients were misclassified. Risk factors for OSA misclassification included age (OR 1.02, 95% CI 1.01–1.03, *P* = 0.001) and body-mass index (BMI) (OR 0.97, 95% CI 0.95–0.99, *P* = 0.015). Risk for misclassification was significant in patients aged ≥ 57 years old, with BMI < 32.3 kg/m^2^. Using total bed time rather than total sleep time to quantify OSA severity was associated with a significant risk of misclassification, particularly in patients aged ≥ 57 years old, with BMI < 32.3 kg/m^2^.

## Introduction

Obstructive sleep apnea (OSA) is a highly prevalent condition with nearly one billion persons affected globally^1^. Untreated OSA results in intermittent hypoxia and is associated with hypertension, stroke, heart failure, diabetes^2^, depression^3^ and gout^4^. It can lead to excessive daytime sleepiness^5^, a reduced quality of life^6^ and an increased risk of road traffic accidents^7^. Morbidity of OSA is related to its severity, which is conventionally measured using the Apnea–Hypopnea Index (AHI). The AHI is computed as the number of apnea and hypopnea events per hour of sleep. Measurement of sleep duration in turn requires electroencephalography, which is part of a full polysomnogram usually done in sleep laboratories. One criticism regarding the use of AHI is its failure to take the duration and depth of respiratory events into account^7^. Although the inclusion of other measures like the Oxygen Desaturation index (ODI) in the evaluation of severity of OSA is emerging^7^, AHI remains the mainstay for OSA diagnosis and severity classification.

Home sleep apnea tests are portable sleep monitoring tests that are cheaper to do than full polysomnograms^8,9^, but lack electroencephalography and thus AHI cannot be computed. Home sleep apnea tests measure the respiratory-event index (REI), calculated as the frequency of apneas and hypopneas divided by the monitoring time. The monitoring time in turn is defined as total recording time minus periods of artifacts and time the patient was awake, as determined by actigraphy, respiratory patterns, or sleep log. In the absence of obvious body movements such as walking, the monitoring time would equate to the total time spent in bed. Bed time is necessarily more than sleep time, with the time taken to fall asleep being the sleep latency, a period that varies considerably between individuals. Given that REI always has a larger denominator than AHI, REI would be smaller than AHI. Using REI would then underestimate the severity of OSA.

Classification of OSA severity is important for treatment choice. Typically, mild OSA is treated conservatively, moderate OSA is treated with mandibular advancement splints or continuous positive airway pressure (CPAP) devices, and severe OSA is treated with CPAP or surgery^10^. Furthermore, severity of OSA is related to adherence to therapy^11^, which may be partially due to response to therapy and partially due to the patient’s motivation to correct a more severe disease state^12^. Therefore, misclassifications of OSA severity could affect both management decisions and outcomes.

Prior studies have demonstrated that portable sleep studies underestimate the severity of OSA compared to full polysomnograms^12^, though the predictors for OSA severity underestimation have not been well studied. We hypothesize that using portable sleep studies in place of full polysomnography leads to significant under diagnosis of OSA and underestimation of OSA severity, and that predictors of OSA severity misclassification exist. In our center, most patients preferred laboratory full polysomnography rather than home-based portable sleep studies due to the availability of subsidies for the former. This situation provided an opportunity to test our hypothesis among a population of patients who would otherwise have qualified for portable sleep studies. By using full polysomnography data, we aimed to describe the potential misclassification of OSA and to investigate the predictors for misclassification. Understanding which patient characteristics increase the risk of misclassification would then help physicians decide on the selection of home sleep studies versus full polysomnograms.

## Methods

Using a retrospective observational design, data were extracted from the sleep laboratory database of National University Hospital (NUH), Singapore, which is a 1200-bed tertiary hospital. This database captures the patient characteristics and results of all adult patients over the age of 18 who underwent sleep studies in NUH. All in-laboratory diagnostic polysomnograms done between January 2014 and March 2017 were included in the analysis. We excluded sleep studies done for therapeutic reasons i.e. titration of continuous positive airway pressure. All polysomnograms were video assisted in an accredited sleep laboratory and were scored manually by certified sleep technicians. The polysomnogram manufacturer was Compumedics**.** All methods were carried out in accordance with relevant guidelines and regulations. Given the non-interventional study design, the Institution Review Board of National Healthcare Group permitted the use of patients’ records for our study (DSRB 2017/00245), and waived the need for informed consent.

The primary outcome of this study was misclassification of OSA. Misclassification of OSA was defined as any OSA severity that was less severe using total bed time (i.e. using REI) versus total sleep time (i.e. using AHI). Sleep apnea events included obstructive, mixed, central and hypopnea events unless otherwise stated. Respiratory effort related arousals were not included. Sleep apnea severity was coded as Normal(0) X < 5; Mild(1) 5 ≤ X < 15; Moderate(2) 15 ≤ X < 30; Severe(3) X ≥ 30, where X represents either REI or AHI. In addition, the use of total sleep time as a denominator was compared to that of total bed time. Paired t-test was used to assess if the difference between the two was significant.

Logistic regression was used to identify predictors of OSA misclassification. Possible predictors tested included the following: age, gender, ethnicity, body mass index (BMI), smoking status, and comorbid conditions, such as diabetes mellitus. Significant predictors were further studied using descriptive statistics and univariate analysis, in order to identify at-risk populations for OSA misclassification who would not be suitable for home sleep apnea tests. Statistical significance was taken as *P* < 0.05.

## Results

**Figure 1:**
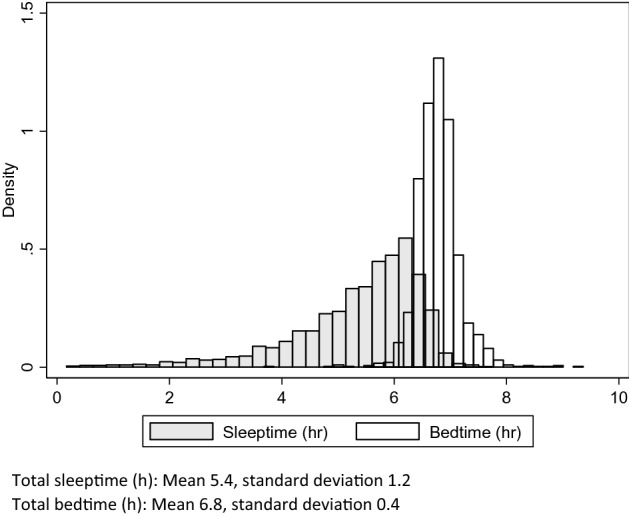
Histograms for total sleep time and bed time. Total sleeptime (h): mean 5.4, standard deviation 1.2. Total bedtime (h): mean 6.8, standard deviation 0.4.

A total of 1621 patients were studied. The mean age was 45.6 ± 15.9 years amongst the patients, of whom 1189 (73.4%) were male. Ethnic composition was similar to previously published data regarding Singapore’s ethnic composition^13^. A majority (92.8%) of the patients were non-smokers. The most common comorbid conditions included diabetes mellitus, hypertension and hyperlipidemia. OSA was the most frequent (98.2%) diagnosis prior to sleep study. Further details regarding the patient characteristics are shown in Table 1. Distributions of total sleep time and total bed time are shown in Fig. 1.

**Table 1:** Patient characteristics and sleep study results.

Patient characteristics	Values
Number of patients	1621
Mean age (years) (SD)	45.6 (15.9)
Male gender (%)	1189 (73.4)
**Ethnicity**	
Chinese (%)	1201 (74.1)
Malay (%)	199 (12.3)
Indian (%)	132 (8.1)
Other (%)	89 (5.5)
Mean height (cm) (SD)	166 (8)
Mean weight (kg) (SD)	82.0 (19.3)
Mean body-mass index (kg/m^2^) (SD)	29.7 (6.6)
**Smoking status**	
Never-smoker (%)	1420 (87.6)
Ex-smoker (%)	84 (5.2)
Current smoker (%)	117 (7.2)
**Comorbid conditions**	
Diabetes mellitus (%)	214 (13.2)
Hypertension (%)	525 (32.4)
Hyperlipidemia (%)	515 (31.8)
Ischemic heart disease (%)	125 (7.7)
Asthma (%)	156 (9.6)
Chronic obstructive pulmonary disease (%)	17 (1.1)
Chronic kidney disease (%)	32 (2.0)
Stroke (%)	52 (3.2)
**Main diagnosis prior to sleep study**	
Obstructive sleep apnea (%)	1592 (98.2)
Obesity hypoventilation syndrome (%)	2 (0.1)
Central sleep apnea (%)	1 (0.1)
Rapid Eye Movement parasomnia (%)	8 (0.5)
Non-rapid eye movement parasomnia (%)	4 (0.3)
Narcolepsy (%)	7 (0.4)
Restless legs syndrome (%)	4 (0.3)
Insomnia (%)	3 (0.2)
**Apnea–hypopnea index**	
< 5/hour (%)	103 (6.4)
5 to < 15/hour (%)	308 (19.0)
15 to < 30/hour (%)	367 (22.6)
30/hour and higher (%)	843 (52.0)

SD: Standard deviation.

A total of 1518(93.6%) patients were diagnosed to have OSA using total sleep time. Substituting total bed time for total sleep time yielded a total of 1479 (91.2%) patients testing positive for OSA. Of note, the total number of misclassifications was 300 (18.5% of the study population), which was statistically significant (*P* < 0.005) (Table 2). Stratifying by each severity grade of OSA, using total sleep instead of total bed time significantly misclassified OSA across all severity grades (Table 3).

**Table 2:** Obstructive sleep apnea classification matrix.

Classification using total bed time	Classification using total sleep time				
	No OSA	Mild OSA	Moderate OSA	Severe OSA	Total
No OSA	103	32	2	5	142
Mild OSA	0	276	96	16	388
Moderate OSA	0	0	269	149	418
Severe OSA	0	0	0	673	673
Total	103	308	367	843	1621

OSA: Obstructive sleep apnea.

Total number of misclassifications = 300/1621 (18.5%).

Pearson chi-square *P* < 0.001.

**Table 3:** Obstructive sleep apnea misclassification.

	Classification using total sleep time	Classification using total bed time	Difference (95% CI)
No OSA (%)	103/1621 (6.4)	142/1621 (8.8)	39/1621 (2.4, 1.7–3.3)
Mild OSA (%)	308/1621 (19.0)	388/1621 (23.9)	80/1621 (4.9, 3.9–6.1)
Moderate OSA (%)	367/1621 (22.6)	418/1621 (25.8)	51/1621 (3.1, 2.4–4.1)
Severe OSA (%)	843/1621 (52.0)	673/1621 (41.5)	170/1621 (10.5, 9.0–12.1)

CI: Confidence interval.

OSA: Obstructive sleep apnea.

With respect to identification of predictors for OSA misclassification, age was identified as a positive predictor for OSA misclassification (OR 1.02, 95% CI 1.01–1.03, *P* = 0.001). Conversely, BMI was a negative predictor for OSA misclassification (OR 0.97, 95% CI 0.95–0.99, *P* = 0.015) (Table 4).Further subgroup analysis using age and BMI quartiles showed the main group driving OSA misclassification was the age group from 57 years old and above, while the BMI group with values of ≥ 32.3 kg/m^2^ was associated with reduced odds of OSA misclassification (Table 5).

**Table 4:** Predictors of obstructive sleep apnea misclassification.

	Univariate OR (95% CI)	Univariate *P* value	Multivariate OR (95% CI)	Multivariate *P* value
Age (years)	1.02 (1.01–1.03)	< 0.001	1.02 (1.01–1.03)	0.001
Male gender	0.72 (0.55–0.95)	0.021	0.79 (0.60–1.04)	0.100
**Ethnicity**				
Chinese	1.02 (0.76–1.35)	0.915	–	–
Malay	0.93 (0.63–1.37)	0.722	–	–
Indian	1.27 (0.82–1.95)	0.286	–	–
Other	0.74 (0.41–1.35)	0.331	–	–
Height (cm)	0.97 (0.96–0.99)	< 0.001	Omitted (collinearity)	–
Weight (cm)	0.99 (0.98–0.99)	< 0.001	Omitted (collinearity)	–
BMI (kg/m^2^)	0.98 (0.96–0.99)	0.016	0.97 (0.95–0.99)	0.015
**Smoking status**				
Non-smoker	1.09 (0.74–1.60)	0.670	–	–
Ex-smoker	0.87 (0.49–1.58)	0.656	–	–
Current	0.96 (0.59–1.57)	0.872	–	–
**Comorbid conditions**				
Diabetes mellitus	1.28 (0.90–1.82)	0.163	–	–
Hypertension	1.51 (1.16–1.95)	0.002	1.24 (0.92–1.67)	0.165
Hyperlipidemia	1.26 (0.97–1.64)	0.082	–	–
IHD	1.50 (0.98–2.31)	0.061	–	–
Asthma	1.26 (0.84–1.88)	0.267	–	–
COPD	1.36 (0.44–4.20)	0.593	–	–
CKD	1.48 (0.66–3.32)	0.342	–	–
Stroke	2.01 (1.10–3.67)	0.023	1.55 (0.83–2.88)	0.169
Non-OSA main diagnosis prior to sleep study	1.70 (0.74–3.87)	0.209	–	–

BMI: Body-mass index.

CKD: Chronic kidney disease.

COPD: Chronic obstructive pulmonary disease.

IHD: Ischemic heart disease.

OSA: Obstructive sleep apnea.

**Table 5:** Misclassification of obstructive sleep apnea, by age and body-mass index quartiles.

	Misclassification (%, 95% CI)	Misclassification OR (95% CI)
1st age quartile (< 33 years)	55/394 (14.0, 10.7–17.8)	Reference
2nd age quartile (33 to < 47 years)	61/413 (14.8, 11.5–18.6)	1.07 (0.72–1.58)
3rd age quartile (47 to < 57 years)	64/366 (17.5, 13.7–21.8)	1.31 (0.88–1.93)
4th age quartile (57 years and older)	120/448 (26.8, 22.7–31.1)	2.25 (1.58–3.21)
1st BMI quartile (< 25.2 kg/m^2^)	85/404 (21.0, 17.2–25.3)	Reference
2nd BMI quartile (25.2 to < 29.6 kg/m^2^)	85/397 (21.4, 17.5–25.8)	1.02 (0.73–1.43)
3rd BMI quartile (29.6 to < 32.3 kg/m^2^)	67/412 (16.3, 12.8–20.2)	0.73 (0.51–1.04)
4th BMI quartile (32.3 kg/m^2^ and higher)	63/408 (15.4, 12.1–19.4)	0.69 (0.48–0.98)
**1st age quartile (< 33 years)**		
With 4th BMI quartile (32.3 kg/m^2^ and higher)	14/104 (13.5, 7.6–21.6)	Reference
With 3rd BMI quartile (29.6 to < 32.3 kg/m^2^)	13/123 (10.6, 5.7–17.4)	0.76 (0.34–1.70)
With 2nd BMI quartile (25.2 to < 29.6 kg/m^2^)	12/65 (18.5, 9.9–30.0)	1.46 (0.63–3.38)
With 1st BMI quartile (< 25.2 kg/m^2^)	16/102 (15.7, 9.2–24.2)	1.20 (0.55–2.60)
**2nd age quartile (33 to < 47 years)**		
With 4th BMI quartile (32.3 kg/m^2^ and higher)	13/109 (11.9, 6.5–19.5)	0.87 (0.39–1.95)
With 3rd BMI quartile (29.6 to < 32.3 kg/m^2^)	17/127 (13.4, 8.0–20.6)	0.99 (0.46–2.12)
With 2nd BMI quartile (25.2 to < 29.6 kg/m^2^)	22/99 (22.2, 14.5–31.7)	1.84 (0.88–3.84)
With 1st BMI quartile (< 25.2 kg/m^2^)	9/78 (11.5, 5.4–20.8)	0.84 (0.34–2.05)
**3rd age quartile (47 to < 57 years)**		
With 4th BMI quartile (32.3 kg/m^2^ and higher)	14/98 (14.3, 8.0–22.8)	1.07 (0.48–2.38)
With 3rd BMI quartile (29.6 to < 32.3 kg/m^2^)	14/85(16.5, 9.4–26.1)	1.27 (0.57–2.83)
With 2nd BMI quartile (25.2 to < 29.6 kg/m^2^)	17/98 (17.4, 10.4–26.3)	1.34 (0.63–2.91)
With 1st BMI quartile (< 25.2 kg/m^2^)	19/85 (22.4, 14.0–32.7)	1.85 (0.87–3.96)
**4th age quartile (57 years and older)**		
With 4th BMI quartile (32.3 kg/m^2^ and higher)	22/97 (22.7, 14.8–32.3)	1.89 (0.90–3.94)
With 3rd BMI quartile (29.6 to < 32.3 kg/m^2^)	23/77(29.9, 20.0–41.4)	2.74 (1.30–5.79)
With 2nd BMI quartile (25.2 to < 29.6 kg/m^2^)	34/135 (25.2, 18.1–33.4)	2.16 (1.09–4.29)
With 1st BMI quartile (< 25.2 kg/m^2^)	41/139 (29.5, 22.1–37.8)	2.69 (1.38–5.26)

BMI: Body-mass index.

CI: Confidence interval.

OR: Odds ratio.

## Discussion

Our study showed that use of total bedtime rather than sleeptime led to significant misclassification of OSA, with both underdiagnosis and with underestimation of severity. Age and BMI were identified as positive and negative predictors of misclassification respectively. In particular, patients who were aged 57 years and older, and who had a BMI < 32.3 kg/m^2^ had increased odds of OSA misclassification.

Our finding is consistent with prior studies that also directly evaluated polysomnogram data^14^. Reasons for this misclassification besides the expectedly higher denominator for total bed time includes the presence of concomitant sleep disturbances such as periodic limb movements of sleep, which may be more common in older persons^12,15^. Such disturbances contribute to lack of sustained sleep, with resultant decreased sleep efficiency. The resultant decrease in total sleep time as compared to total bed time leads to a larger discrepancy between AHI scores calculated using the two. Although our study population did not have any patients diagnosed with movement disorders, 246 patients (15.2% of our cohort) had a periodic limb movement index > 5 (using sleep time). The odds of OSA misclassification increased with every 1 unit increase in periodic limb movement index (odds ratio 1.01, 95% CI 1.00–1.03, *P* = 0.014), when adjusted for age.

One reason why misclassification is independently associated with increasing age could be that in older patients sleep latency can be increased^16^. In addition, increasing age is associated with decreasing ability to maintain sleep as well^17^. These findings were attributed in previous studies to be due to the overlap of increasing age with increasing medical and psychiatric disorders and related health burdens^18^. These two factors contribute to a decrease in the ratio of total sleep time to total bed time, hence accounting for how increasing age may be associated with increased risk of misclassification.

Regarding the inverse association of misclassification with increasing BMI, this is explained by the raised respiratory arousal threshold in patients with increased BMI^19^. Of note there are different cut of points for obesity in Asian populations, with BMI values of ≥ 23 taken to be overweight^20^. In obese patients, there is a higher respiratory arousal threshold which protects against interrupted sleep. Conversely, in non-obese patients, this protective effect is lost. There would therefore be more interrupted sleep in the non-obese cohort with reduction in the ratio of total sleep time to total bed time, thereby explaining how increasing BMI is inversely associated with the risk of misclassification. Indeed, from our data, arousal index decreased by − 0.221 (95% CI − 0.341 to − 0.101, *P* < 0.001) for every 1 unit increase in BMI, adjusted for AHI (using sleeptime).

A strength of our study is that our study population consists of a multiethnic population which is representative of the diverse ethnicities present in Asia. This allows for our study to be applied in other healthcare settings across Asia and other populations with similar diversity. Furthermore, our study has gone on to evaluate for possible predictors for misclassification, which potentially may allow for clinicians to better select for patients who may be unsuitable for home sleep studies. In addition, as virtually all of our patients in the NUH sleep laboratory had in-lab sleep studies performed due to financial subsidies (compared to no subsidies for home sleep studies), we were able to comprehensively capture full polysomnographic data from a complete population of patients with suspected sleep disorders.

A limitation of our study is our lack of data on other comorbidities, in particular psychiatric comorbidities, which have been found previously to be contributory to poor sleep^18^. It is possible that there may be other, unidentified comorbidities that could have contributed to misclassification of OSA as well. It could also be argued that a limitation of in-lab sleep studies includes a possible adverse effect on sleep quality, owing to the unfamiliar environment^21^. Sleep latency and quality could be affected, which may result in a larger discordance between total bed time and sleep time. However, a prospective randomised study showed there was no evidence of a better quality of sleep and recording tolerance at home^22^, which reduces the impact that this would have on our study. Another limitation of our study is its retrospective design, which may be a possible source of bias.

The clinical implications of our study would be that the use of home sleep apnea testing needs to be done with caution, especially in a patient population at high pretest risk of moderate OSA, as misclassification may result in the appropriate therapies not being offered. Patients may also be lulled into thinking their OSA is less severe than it really is, and hence less motivated to adhere to therapy. Full polysomnography may be preferred in the population of age ≥ 57 years old with BMI < 32.3 given the presence of identified predictors of OSA misclassification.

Future research in this field could delve into affirming the results of this study with a randomised control trial of older patients with lower BMI undergoing either home sleep test or polysomnography. Other aspects to consider will include looking into how processes can be changed in the sleep clinic to flag up patients who may not be suitable for home sleep apnea testing. This study can also stimulate device manufacturers to innovate and create ways to more accurately detect sleep in home sleep devices^23^. A possibility is to include a user controlled device which the user can use to signify the number of awakenings overnight, to better estimate the actual sleep time.

To conclude, use of total sleep time for calculation of AHI^24^ may be associated with significant misclassification of OSA and underestimation of severity, which in turn may affect treatment. Predictors of misclassification included increasing age as well as lower BMI.

## References

[1] Benjafield AV, Ayas NT, Eastwood PR, Heinzer R, Ip MSM, Morrell MJ, Nunez CM, Patel SR, Penzel T, Pepin JL, Peppard PE, Sinha S, Tufik S, Valentine K, Malhotra A (2019). Estimation of the global prevalence and burden of obstructive sleep apnoea: A literature-based analysis. Lancet Respir. Med..

[2] Pinto JA, Ribeiro DK, Cavallini AF, Duarte C, Freitas GS (2016). Comorbidities associated with obstructive sleep apnea: A retrospective study. Int. Arch. Otorhinolaryngol..

[3] Osman AM, Carter SG, Carberry JC, Eckert DJ (2018). Obstructive sleep apnea: current perspectives. Nat. Sci. Sleep.

[4] van Durme C, Spaetgens B, Driessen J (2020). Obstructive sleep apnea and the risk of gout: A population-based case-control study. Arthritis Res. Ther..

[5] Vernet C, Redolfi S, Attali V (2011). Residual sleepiness in obstructive sleep apnoea: Phenotype and related symptoms. Eur. Respir. J..

[6] Isidoro SI, Salvaggio A, Lo Bue A (2015). Effect of obstructive sleep apnea diagnosis on health related quality of life. Health Qual. Life Outcomes.

[7] Temirbekov D, Güneş S, Yazıcı ZM, Sayın İ (2018). The ignored parameter in the diagnosis of obstructive sleep apnea syndrome: The oxygen desaturation index. Turk. Arch. Otorhinolaryngol..

[8] Rosen CL, Auckley D, Benca R (2012). A multisite randomized trial of portable sleep studies and positive airway pressure autotitration versus laboratory-based polysomnography for the diagnosis and treatment of obstructive sleep apnea: The HomePAP study. Sleep.

[9] Kim RD, Kapur VK, Redline-Bruch J (2015). An economic evaluation of home versus laboratory-based diagnosis of obstructive sleep apnea. Sleep.

[10] Veasey SC, Rosen IM (2019). Obstructive sleep apnea in adults. N. Engl. J. Med..

[11] Jacobsen AR, Eriksen F, Hansen RW, Erlandsen M, Thorup L, Damgard MB, Kirkegaard MG, Hansen KW (2017). Determinants for adherence to continuous positive airway pressure therapy in obstructive sleep apnea. PLoS ONE.

[12] Bianchi MT, Goparaju B (2017). Potential underestimation of sleep apnea severity by at-home kits: Rescoring in-laboratory polysomnography without sleep staging. J. Clin. Sleep Med..

[13] Gov.sg. *What Are The Racial Proportions Among Singapore Citizens?* (accessed 18 January 2021, 2021) https://www.gov.sg/article/what-are-the-racial-proportions-among-singapore-citizens.

[14] Yang EH, Hla KM, McHorney CA, Havighurst T, Badr MS, Weber S (2000). Sleep apnea and quality of life. Sleep.

[15] Doan TT, Koo BB, Ogilvie RP, Redline S, Lutsey PL (2018). Restless legs syndrome and periodic limb movements during sleep in the Multi-Ethnic Study of Atherosclerosis. Sleep.

[16] Suzuki K, Miyamoto M, Hirata K (2017). Sleep disorders in the elderly: Diagnosis and management. J. Gen. Fam. Med..

[17] Li J, Vitiello MV, Gooneratne NS (2018). Sleep in normal aging. Sleep Med. Clin..

[18] Vitiello MV, Moe KE, Prinz PN (2002). Sleep complaints cosegregate with illness in older adults: Clinical research informed by and informing epidemiological studies of sleep. J. Psychosom. Res..

[19] Gray EL, McKenzie DK, Eckert DJ (2017). Obstructive sleep apnea without obesity is common and difficult to treat: Evidence for a distinct pathophysiological phenotype. J. Clin. Sleep Med..

[20] Pan WH, Yeh WT (2008). How to define obesity? Evidence-based multiple action points for public awareness, screening, and treatment: An extension of Asian-Pacific recommendations. Asia Pac. J. Clin. Nutr..

[21] Bruyneel M, Libert W, Ameye L, Ninane V (2015). Comparison between home and hospital set-up for unattended home-based polysomnography: A prospective randomized study. Sleep Med..

[22] Portier F, Portmann A, Czernichow P, Vascaut L, Devin E, Benhamou D, Cuvelier A, Muir JF (2000). Evaluation of home versus laboratory polysomnography in the diagnosis of sleep apnea syndrome. Am. J. Respir. Crit. Care Med..

[23] Penzel T, Schöbel C, Fietze I (2018). New technology to assess sleep apnea: Wearables, smartphones, and accessories. F1000Res.

[24] Krishnaswamy U, Aneja A, Kumar RM, Kumar TP (2015). Utility of portable monitoring in the diagnosis of obstructive sleep apnea. J. Postgrad. Med..

